# Analysis of the Optimum Usage of Slag for the Compressive Strength of Concrete

**DOI:** 10.3390/ma8031213

**Published:** 2015-03-18

**Authors:** Han-Seung Lee, Xiao-Yong Wang, Li-Na Zhang, Kyung-Taek Koh

**Affiliations:** 1 Department of Architectural Engineering, Hanyang University, Ansan-Si 425-791, Korea; E-Mail: ercleehs@hanyang.ac.kr; 2 Department of Architectural Engineering, Kangwon National University, Chuncheon-Si 200-701, Korea; E-Mail: lnzhangbeibei@outlook.com; 3 Structural Engineering Research Division, Korea Institute of Construction Technology, Goyang-Si 411-712, Korea; E-Mail: ktgo@kict.re.kr

**Keywords:** slag, concrete, optimum usage, compressive strength, hydration model

## Abstract

Ground granulated blast furnace slag is widely used as a mineral admixture to replace partial Portland cement in the concrete industry. As the amount of slag increases, the late-age compressive strength of concrete mixtures increases. However, after an optimum point, any further increase in slag does not improve the late-age compressive strength. This optimum replacement ratio of slag is a crucial factor for its efficient use in the concrete industry. This paper proposes a numerical procedure to analyze the optimum usage of slag for the compressive strength of concrete. This numerical procedure starts with a blended hydration model that simulates cement hydration, slag reaction, and interactions between cement hydration and slag reaction. The amount of calcium silicate hydrate (CSH) is calculated considering the contributions from cement hydration and slag reaction. Then, by using the CSH contents, the compressive strength of the slag-blended concrete is evaluated. Finally, based on the parameter analysis of the compressive strength development of concrete with different slag inclusions, the optimum usage of slag in concrete mixtures is determined to be approximately 40% of the total binder content. The proposed model is verified through experimental results of the compressive strength of slag-blended concrete with different water-to-binder ratios and different slag inclusions.

## 1. Introduction

Slag is a by-product obtained during steel manufacturing and is commonly used in concrete because it improves durability and reduces porosity by improving the interface with the aggregate. Economic and ecologic benefits in the form of energy-savings and resource-conserving properties can also be achieved using slag-blended cement [1,2].

Compressive strength is the most important engineering property of concrete. To ensure progress in construction and safety in engineering practices, we aim to develop understanding on the strength development of concrete. Many experimental investigations have been conducted on the strength development of slag-blended concrete. The strength development of slag-blended concrete closely relates to the water-to-binder ratio, slag replacement ratio, and curing conditions. Beushausen *et al.* [3] found that, under moist curing conditions and when the slag replacement ratio is less than 50%, the 1-day early-age strength of concrete almost linearly decreases with the increase in the slag replacement ratio. At the ages of 28 and 56 days, due to the formation of calcium silicate hydrate (CSH) from the slag reaction, the compressive strength of slag-blended concrete can surpass that of control Portland cement concrete. Shariq *et al.* [4] found that, for concrete incorporating larger slag content (higher than 60% of the binder content), until the age of 180 days, the compressive strength of slag-blended concrete is still lower than that of Portland cement concrete. Oner and Akyuz [5] systematically investigated the effect of slag inclusions on the compressive strength development of concrete. They found that at a late age of 365 days, compressive strength of concrete mixtures containing slag increases as the amount of slag increases. After an optimum point of slag, a further increase in slag no longer improves the compressive strength.

To theoretically deduce the optimum usage of slag, models for compressive strength development of slag-blended concrete are necessary. Compared with abundant experimental studies [3,4,5], theoretical analysis of the compressive strength development of slag-blended concrete is limited. For comparing the relative performance of various supplementary cementing materials (SCMs: silica fume, fly ash, slag, natural pozzolans, *etc.*) as regards Portland cement, Papadakis [6,7] proposed an efficiency factor of SCMs that can be considered as equivalent to Portland cement. However, it should be noted that Papadakis’ model [6,7] does not consider the effects of curing age and slag replacement ratios on the efficiency factor. Using a blended hydration model considering both cement hydration and slag reaction, De Schutter [8,9] evaluated the early-age strength development of hardening slag-blended concrete. For late-age concrete, due to the significantly decreasing heat evolution rate, it is difficult to use De Schutter’s model to evaluate the degree of hydration and strength development [2]. In addition, when the slag replacement ratio and water-to-binder ratio change, the coefficients of De Schutter’s model will vary [2]. Using an artificial neural network, Bilim [10] evaluated the early-age strength and late-age strength of slag-blended concrete with different water-to-binder ratios and slag replacement ratios. However, we should note that artificial neural networks are a type of numerical regression method. Many parameters are necessary to build the input layer and hidden layers of artificial neural networks. The physical meaning of these parameters is not clear. Hence, it is difficult to adopt current models [5,6,7,8,9,10] to evaluate the strength development of slag-blended concrete with different mixing proportions. Moreover, current models [5,6,7,8,9,10] cannot be used to analyze the optimum usage of slag in concrete mixtures.

To overcome the weaknesses of the current research [5,6,7,8,9,10], this paper presents a numerical procedure to simulate the cement hydration, slag reaction, microstructure and strength development of hardening slag-blended concrete. The properties of concrete are determined considering contributions from cement hydration and slag reaction. Using parameter analysis of the compressive strength development of concrete with different slag inclusions, the optimum usage of slag in concrete mixtures is determined.

The innovations of this research are as follows. First, the proposed numerical procedure is valid for concrete with various mixing proportions, such as different water-to-binder ratios or different slag replacement ratios. The dependences of cement and slag reactivity on concrete mixing proportions and curing conditions are clarified; Second, the proposed numerical procedure is valid for both early-age concrete and late-age concrete. Evolutions of concrete properties are expressed as functions of reaction degrees of cement and slag; Third, the proposed numerical procedure evaluates the macro properties, such as the compressive strength of concrete, by using the microstructures of concrete such as the CSH content and phase volume fraction. The physical meanings of parameters in the proposed model are much clearer than those used in the artificial neural network model [10].

## 2. Hydration Model of Cement–Slag Blends

### 2.1. Hydration Model of Portland Cement

Tomosawa [11] proposed a shrinking-core model for the hydration of Portland cement. This model is expressed as a single equation consisting of three coefficients: $k_{d}$, the reaction coefficient in the induction period; $D_{e}$, the effective diffusion coefficient of water through the C–S–H gel; and $k_{ri}$, a coefficient of the reaction rate of the mineral compound *i* of cement, as shown in Equations (1-1) and (1-2) below:

(1-1) $$\frac{d{\text{α}}_{i}}{dt}=\frac{3\left(S_{w}/S_{0}\right){\text{ρ}}_{w}C_{w-free}}{(v+w_{g})r_{0}{\text{ρ}}_{c}}\frac{1}{(\frac{1}{k_{d}}-\frac{r_{0}}{D_{e}})+\frac{r_{0}}{D_{e}}{\left(1-{\text{α}}_{i}\right)}^{\frac{-1}{3}}+\frac{1}{k_{ri}}{\left(1-{\text{α}}_{i}\right)}^{\frac{-2}{3}}}$$

(1-2) $$\text{α}=\frac{\sum_{i=1}^{4}{\text{α}}_{i}g_{i}}{\sum_{i=1}^{4}g_{i}}$$

where ${\text{α}}_{i}$ (*i* = 1, 2, 3, and 4) represents the reaction degree of the cement mineral compounds C_3_S, C_2_S, C_3_A, and C_4_AF, respectively; α is the degree of cement hydration and can be calculated from the weight fraction of mineral compound $g_{i}$ and reaction degree of mineral compound ${\text{α}}_{i}$; ν is the stoichiometric ratio of the mass of water to cement (=0.25); $w_{g}$ is the physically bound water in C–S–H gel (=0.15); ${\text{ρ}}_{w}$ is the density of water; ${\text{ρ}}_{c}$ is the density of the cement; $C_{w-free}$ is the amount of water at the exterior of the C–S–H gel; $r_{0}$ is the radius of unhydrated cement particles; $S_{w}$ is the effective surface area of the cement particles in contact with water; and $S_{0}$ is the total surface area if the surface area develops unconstrained.

The reaction coefficient $k_{d}$ is assumed to be a function of the degree of hydration, as shown in Equation (2), where *B* and *C* are the coefficients determining this factor; *B* controls the rate of the initial shell formation, and *C* controls the rate of the initial shell decay.

(2) $$k_{d}=\frac{B}{{\text{α}}^{1.5}}+C{\text{α}}^{3}$$

The effective diffusion coefficient of water is affected by the tortuosity of the gel pores as well as the radii of the gel pores in the hydrate. This phenomenon can be described as a function of the degree of hydration and is expressed as follows:

(3) $$D_{e}=D_{e0}\;\text{ln}(\frac{1}{\text{α}})$$

In addition, free water in the capillary pores is depleted as hydration of cement minerals progresses. Some water is bound in the gel pores, and this water is not available for further hydration, an effect that must be taken into consideration in every step of the progress of the hydration. Therefore, the amount of water in the capillary pores, $C_{w-free}$, is expressed as a function of the degree of hydration in the previous step, as shown in Equation (4).

(4) $$C_{w-free}=\frac{W_{0}-0.4*\text{α}*C_{0}}{W_{0}}$$

where $C_{0}$ and $W_{0}$ are the mass fractions of cement and water in the mix proportion.

The effect of temperature on these reaction coefficients is assumed to follow Arrhenius’s law as shown in Equations (5)–(8):

(5) $$B=B_{20}\;\text{exp}(-{\text{β}}_{1}(\frac{1}{T}-\frac{1}{293}))$$

(6) $$C=C_{20}\;\text{exp}(-{\text{β}}_{2}(\frac{1}{T}-\frac{1}{293}))$$

(7) $$k_{ri}=k_{ri20}\;\text{exp}(-\frac{E}{R}(\frac{1}{T}-\frac{1}{293}))$$

(8) $$D_{e}=D_{e20}\;\text{exp}(-{\text{β}}_{3}(\frac{1}{T}-\frac{1}{293}))$$

where ${\text{β}}_{1}$, ${\text{β}}_{2}$, E/R, and ${\text{β}}_{3}$ are temperature sensitivity coefficients and $B_{20}$, $C_{20}$, $k_{ri20}$, and $D_{e20}$ are the values of B, C, $k_{ri}$, and $D_{e}$ at 20 °C.

On the basis of the degree of reaction of the mineral compounds of cement [12], the parameters of the hydration model are calibrated and shown in Table 1. Using this Portland cement hydration model, Tomosawa [11] evaluated the heat evolution rate, adiabatic temperature rise, compressive strength development, and thermal stress development in both ordinary strength concrete and high strength concrete. However, it should be noted that Tomosawa’s model is valid only for Portland cement. For slag-blended cement, due to the coexistence of Portland cement hydration and the chemical reaction of slag, Tomosawa’s model is not valid. To model the hydration of slag-blended concrete, the reaction model of slag should be built and the mutual interactions between cement hydration and slag reaction should be clarified.

**Table 1:** Coefficients of the cement hydration model.

$B_{20}$ (cm/h)	*C*_20_ (cm/h)	$k_{rC_{3}S20}$ (cm/h)	$k_{rC_{2}S20}$ (cm/h)	$k_{rC_{3}A20}$ (cm/h)	$k_{rC_{4}AF20}$ (cm/h)	$D_{e20}$ (cm^2^/h)	${\text{β}}_{1}$ (K)	${\text{β}}_{2}$ (K)	${\text{β}}_{3}$ (K)	$\frac{E}{R}$ (K)
8.09 × 10^−9^	0.02	9.03 × 10^−6^	2.71 × 10^−7^	1.35 × 10^−6^	6.77 × 10^−8^	8.62 × 10^−10^	1000	1000	7500	5400

### 2.2. Slag Reaction Model

The hydration rate of slag depends on the amount of calcium hydroxide in the hydrating cement-slag blends and the reaction degree of the mineral admixtures [8,11,12,13]. Compared with silica fume, the hydration rate of slag is much lower due to the larger particle size. Similar to the hydration process of cement, the reaction of slag can be divided into three processes: an initial dormant period, the phase-boundary reaction and diffusion processes [8,13]. By considering these points, we originally proposed that the reaction equation of slag can be written as follows:

(9-1) $$\frac{d{\text{α}}_{SG}}{dt}=\frac{CH(t)}{P}\frac{W_{cap}}{W_{0}}\frac{3{\text{ρ}}_{w}}{v_{SG}r_{SG0}{\text{ρ}}_{SG}}\frac{1}{(\frac{1}{k_{dSG}}-\frac{r_{SG0}}{D_{eSG}})+\frac{r_{SG0}}{D_{eSG}}{\left(1-{\text{α}}_{SG}\right)}^{\frac{-1}{3}}+\frac{1}{k_{rSG}}{\left(1-{\text{α}}_{SG}\right)}^{\frac{-2}{3}}}$$

(9-2) $$k_{dSG}=\frac{B_{SG}}{{\left({\text{α}}_{SG}\right)}^{1.5}}+C_{SG}*{\left({\text{α}}_{SG}\right)}^{3}$$

(9-3) $$D_{eSG}=D_{eSG0}*\text{ln}(\frac{1}{{\text{α}}_{SG}})$$

where ${\text{α}}_{SG}$ is the degree of the reaction of slag; CH(t) is the calcium hydroxide mass in a unit volume of hydrating cement-slag blends; P is the mass of slag in the mixture proportion; $W_{cap}$ is the mass of capillary water; $v_{SG}$ is the stoichiometric ratio of the mass of CH to slag; $r_{SG0}$ is the radius of a slag particle; ${\text{ρ}}_{SG}$ is the density of the slag; $k_{dSG}$ is the reaction rate coefficient in the dormant period ($B_{SG}$ and $C_{SG}$ are coefficients); $D_{eSG0}$ is the initial diffusion coefficient; and $k_{rSG}$ is the reaction rate coefficient. Slag shows both cementitious behavior (latent hydraulic activity) and pozzolanic characteristics (reaction with lime). In Equation (9-1), the term $\frac{CH(t)}{P}$ considers the pozzolanic characteristics of slag, and the term $\frac{W_{cap}}{W_{0}}$ considers the latent hydraulic activity of slag.

The influence of temperature on the slag reaction is originally considered by the Arrhenius law as follows:

(9-4) $$B_{SG}=B_{SG20}\;\text{exp}(-{\text{β}}_{1SG}(\frac{1}{T}-\frac{1}{293}))$$

(9-5) $$C_{SG}=C_{SG20}\;\text{exp}(-{\text{β}}_{2SG}(\frac{1}{T}-\frac{1}{293}))$$

(9-6) $$D_{eSG0}=D_{eSG20}\;\text{exp}(-{\text{β}}_{3SG}(\frac{1}{T}-\frac{1}{293}))$$

(9-7) $$k_{rSG}=k_{rSG20}\;\text{exp}(-\frac{E_{SG}}{R}(\frac{1}{T}-\frac{1}{293}))$$

where $B_{SG20}$, $C_{SG20}$, $D_{eSG20}$, and $k_{rSG20}$ are the values of $B_{SG}$, $C_{SG}$, $D_{eSG0}$, and $k_{rSG}$ at 293 K, respectively, and ${\text{β}}_{1SG}$, ${\text{β}}_{2SG}$, ${\text{β}}_{3SG}$, and $E_{SG}/R$ are the temperature sensitivity coefficients of $B_{SG}$, $C_{SG}$, $D_{eSG0}$, and $k_{rSG}$, respectively. The temperature sensitivity coefficients of slag can be determined from the reaction degree of slag at different curing temperatures [11,14].

### 2.3. Interactions between Cement Hydration and Slag Reaction

Based on analysis of the experimental results of the amount of chemically bound water, adiabatic temperature rise, and temperature measurement of small quasi-adiabatic blocks, Maekawa *et al.* [14] stated that the reaction of slag can be roughly described by the following approximate key figures:

$$\begin{array}{ll} \text{Calcium}\;\text{hydroxide} & 0.22\;\text{g}/\text{g}\;\text{slag}\\ \text{Chemically}\;\text{bound}\;\text{water} & 0.30\;\text{g}/\text{g}\;\text{slag}\\ \text{Gel}\;\text{water} & 0.15\;\text{g}/\text{g}\;\text{slag} \end{array}$$

Using the hydration degree of cement, reaction degree of slag, and stoichiometry of the reaction of slag [14], the amounts of calcium hydroxide, chemically bound water, and capillary water in cement-slag blends during hydration can be originally determined with the following equations:

(10) $$CH(t)=RCH_{CE}*C_{0}*\text{α}-0.22*{\text{α}}_{SG}*P$$

(11) $$W_{cap}=W_{0}-0.4*C_{0}*\text{α}-0.30*{\text{α}}_{SG}*P-0.15*{\text{α}}_{SG}*P$$

(12) $$W_{cbm}=v*C_{0}*\text{α}+0.3*{\text{α}}_{SG}*P$$

In Equation (10), $RCH_{CE}$ is the mass of produced calcium hydroxide from the hydration of cement. In Equation (12), $W_{cbm}$ is the mass of chemically bound water. As shown in Equations (10)–(12), the evolution of calcium hydroxide, chemically bound water, and capillary water in cement-slag blends depends on both cement hydration and slag reaction.

As proposed by Papadakis [6,7], for slag-blended concrete, the calcium silicate hydrate (CSH) content, which is the most critical parameter in strength development, can be calculated as a function of the cement content, the slag content, the weight fraction of silica in cement $f_{S,C}$ and slag $f_{S,P}$, and the weight fraction of the reactive oxide SiO_2_ in the slag ${\text{γ}}_{S}$. Combining Papadakis’ chemical reaction equation [6,7] and the hydration reaction Equations (1) and (9), the amount of CSH in hardening slag-blended concrete can be initially calculated as follows:

(13) $$CSH(t)=2.85(f_{S,C}*C_{0}*\text{α}+{\text{γ}}_{s}*f_{S,P}*P*{\text{α}}_{SG})$$

When slag is incorporated into concrete, two possible reasons may be adopted to explain the change in the hydration process. One is the chemical reaction of amorphous phases in slag, and the other is the influence of slag on the hydration of cement. In the current paper, the new model that is originally proposed can describe the reaction of slag. The influence of slag on the hydration of cement is originally considered through the amount of capillary water (Equation (11)) and the dilution effect (Equation (4)) [14]. Hence, the proposed model shows a strong ability to simulate the hydration of concrete containing slag. Furthermore, the development of properties of hardening slag-blended concrete can be evaluated based on the degree of the reactions of cement and slag.

### 2.4. Calibration of the Reaction Coefficients of the Slag Reaction Model

Iyoda *et al.* [15] investigated the reaction degrees of slag in cement-slag paste considering the effects of curing temperatures (5, 20 and 40 °C) and slag replacement ratios (42% and 67% mass fractions). The water-to-binder ratio of cement-slag paste is 0.5. At the ages of 1, 3, 7, 28, 56, and 91 days, the reaction degree of slag was measured using a selective dissolution method. The chemically bound water was measured using an ignition loss method.

For each curing temperature, 5, 20 and 40 °C, the reaction coefficients of slag, $B_{SG}$, $C_{SG}$, $D_{eSG}$, and $k_{rSG}$, can be calibrated through experimental results of the reaction degrees of slag. Furthermore, the temperature sensitivity coefficients of $B_{SG}$, $C_{SG}$, $D_{eSG}$, and $k_{rSG}$ can be determined using the reaction coefficients at different curing temperatures. The values of the slag reaction coefficients and the temperature sensitivity coefficients are shown in Table 2. These fitted parameters for slag are not changed from one mix to another. When the water-to-binder ratio or slag replacement is changed, these parameters of slag do not vary.

**Table 2:** Coefficients of the slag reaction model.

$B_{20SG}$ (cm/h)	CSG20 (cm/h)	$k_{rSG20}$ (cm/h)	$D_{eSG20}$ (cm^2^/h)	${\text{β}}_{1SG}$ (K)	${\text{β}}_{2SG}$ (K)	${\text{β}}_{3SG}$ (K)	$\frac{E_{SG}}{R}$ (K)
8.93 × 10^−9^	0.1	1.0 × 10^−5^	1.86 × 10^−9^	1000	1000	5000	7000

**Figure 1:**
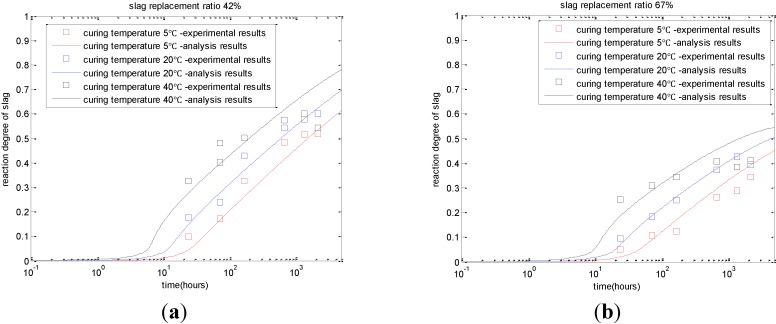
Reaction degree of slag with different slag replacement ratios and curing temperatures (experimental results are taken from reference [15]). (**a**) cement-slag paste with 42% slag; (**b**) cement-slag paste with 67% slag.

**Figure 2:**
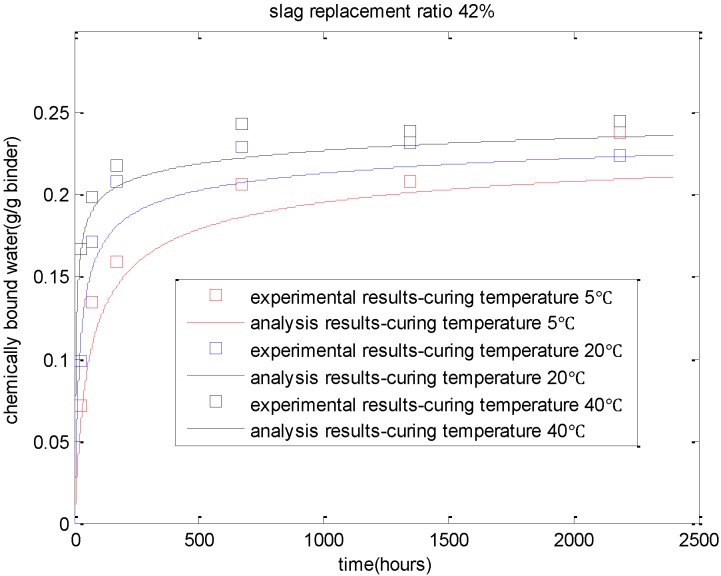
Evaluation of chemically bound water of cement-slag blends (water-to-binder ratio of 0.42) (experimental results are taken from reference [15]).

As shown in Figure 1, with the increase in curing temperature, the reaction degree of slag increases correspondingly. In addition, when the replacement ratio of slag increases from 42% (Figure 1a) to 67% (Figure 1b), due to the shortage of calcium hydroxide, the reaction degrees of slag will decrease significantly. As shown in Figure 2, due to the increasing curing temperatures, the cement hydration and slag reaction will accelerate. Given a certain age, more chemically bound water will be produced for paste with a higher curing temperature. For cement-slag paste cured at 40 °C, the increment of chemically bound water content is marginal after the age of 28 days because of the decreasing reaction rate of cement and slag.

**Figure 3:**
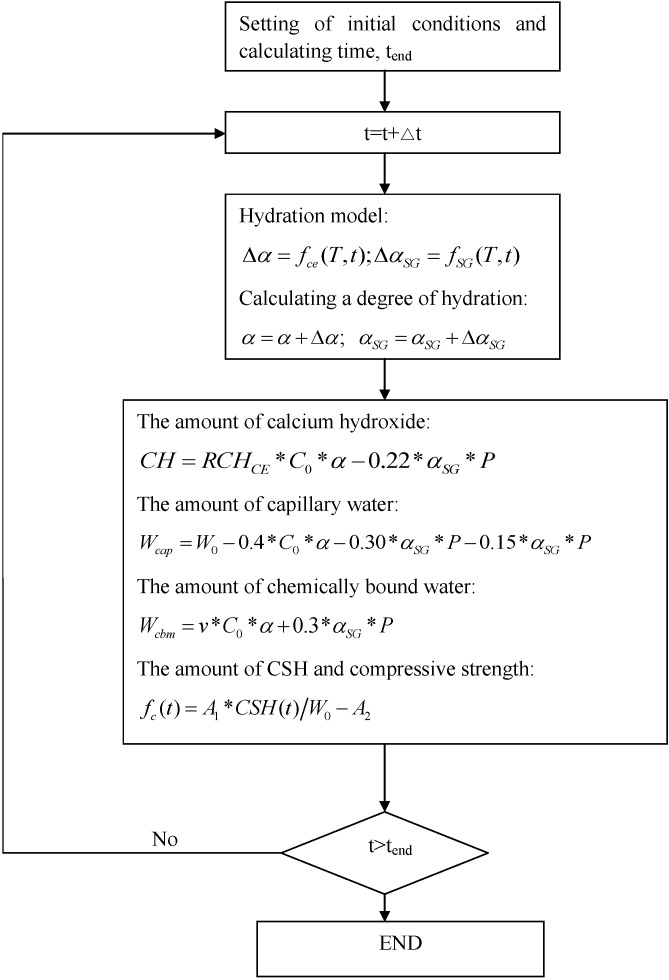
Flowchart of modeling.

The flow chart of modeling is summarized in Figure 3. At each time step, the cement hydration degree and slag reaction degree are calculated. The calcium hydroxide contents, capillary water contents, and chemically bound water contents are determined considering contributions from cement hydration and slag reaction. Furthermore, by using the reaction degrees of cement and slag, the phase volume fractions, calcium silicate hydrate content [16], and strength development of hardening concrete can be determined.

## 3. Analysis of Optimum Usage of Slag for the Compressive Strength of Concrete

As reported by Papadakis [6,7], the compressive strength of concrete is mainly dependent on the amount of calcium silicate hydrate (CSH). For hardening slag-blended concrete, as shown in Equation (13), the amount of CSH relates to both the cement hydration and slag reaction. As proposed by Neville [17], the relation between the compressive strength of concrete and CSH contents can be described by a linear equation as follows:

(14) $$f_{c}(t)=A_{1}*\frac{CSH(t)}{W_{0}}-A_{2}$$

where $f_{c}(t)$ is the compressive strength of concrete, and $A_{1}$ and $A_{2}$ are coefficients related to compressive strength. As shown in this equation, for hardening concrete, the compressive strength development starts after a threshold degree of hydration. When the degree of hydration is less than this threshold degree of hydration, the compressive strength of concrete is zero. The concept of threshold degree of hydration is similar to that of the final setting time of concrete (final set means complete solidification and the beginning of hardening, and in concrete technology, hardening is the phenomenon of strength gain with time [1]).

The experimental results of Cheng *et al.* [18] are used to verify the proposed model. In the study, three water/binder ratios (0.35, 0.50, and 0.70) and three substitution ratios of cement with slag (10%, 20%, and 40%) were selected for the preparation of concrete specimens. The cement used is ASTM type I cement, the fineness of the granulated blast furnace slag is 4000 cm^2^/g, the maximum size of the coarse aggregate is 20 mm, and the fineness modulus of the fine aggregate is 2.96. The chemical compositions of cement and slag are shown in Table 3 and Table 4, respectively. The mixture proportions of concrete are shown in Table 5. The size of the cylinder specimen for the compression test is 100 × 200 mm. The specimens were tested at five ages (1, 3, 7, 28, and 56 days) for the compressive strength measurement.

**Figure 4:**
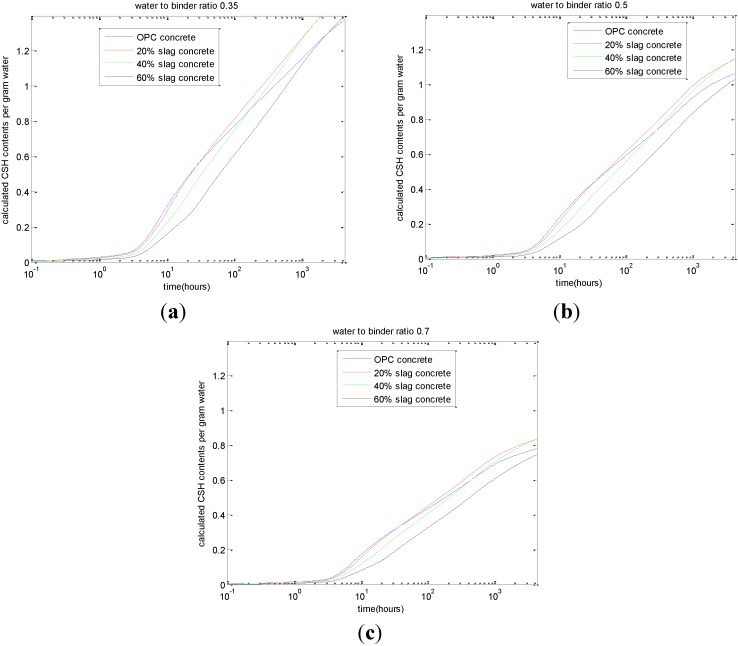
Calculated calcium silicate hydrate (CSH) content. (**a**) water-to-binder ratio of 0.35; (**b**) water-to-binder ratio of 0.5; (**c**) water-to-binder ratio of 0.7.

Using the blended cement hydration model, the amount of CSH can be calculated and is shown in Figure 4. As shown in this figure, for early-age slag-blended concrete, the produced CSH contents are less than that of control concrete, while for late-age slag-blended concrete, the CSH contents can surpass that of the control Portland cement concrete. With the increase in the slag replacement ratio from 0.2 to 0.6, the age corresponding to the surpassing of CSH will be postponed. Alternately, based on the calculated CSH contents and measured compressive strength of concrete, the strength coefficients of Equation (14) can be calibrated. The values of *A*_1_ for a water-to-binder ratio of 0.7, 0.5, and 0.35 are 52.39, 61.54, and 55.37, respectively. The values of *A*_2_ for a water-to-binder ratio of 0.7, 0.5, and 0.35 are 9.81, 12.04, and 4.47, respectively.

**Table 3:** Chemical composition of Portland cement [18].

Chemical Composition (mass %)							Blaine (cm^2^/g)
SiO_2_	Al_2_O_3_	Fe_2_O_3_	CaO	MgO	SO_3_	L.O.I	
20.6	4.0	6.1	62.8	2.6	3.1	0.8	3090

**Table 4:** Chemical composition of slag [18].

Chemical Composition (mass %)									Blaine (cm^2^/g)
SiO_2_	Al_2_O_3_	Fe_2_O_3_	CaO	MgO	SO_3_	Na_2_O	K_2_O	L.O.I	
34.4	9.0	2.58	44.8	4.43	2.26	0.62	0.5	1.32	4000

**Table 5:** Mixing proportions of concrete containing slag [18].

	Water-to-Binder Ratio	Slag Replacement Ratio	Water (kg/m^3^)	Cement (kg/m^3^)	Slag (kg/m^3^)	Sand (kg/m^3^)	Aggregate (kg/m^3^)	Water Reducing Agent (Binder ×%)
WB35	0.35	-	202.8	591	0	570	973	4.1
WB35-10	0.35	10%	202.8	532	59	565	973	4.1
WB35-20	0.35	20%	202.8	473	118	560	973	4.1
WB35-40	0.35	40%	202.8	355	236	552	973	4.1
WB50	0.5	-	206.5	414	0	718	973	0.4
WB50-10	0.5	10%	206.5	372	41	715	973	0.4
WB50-20	0.5	20%	206.5	331	83	712	973	0.4
WB50-40	0.5	40%	206.5	248	165	706	973	0.4
WB70	0.7	-	206.9	296	0	815	973	0
WB70-10	0.7	10%	206.9	266	29	815	973	0
WB70-20	0.7	20%	206.9	237	59	812	973	0
WB70-40	0.7	40%	206.9	177	118	807	973	0

At the macroscopic level, concrete is a composite material consisting of discrete aggregates dispersed in a continuous cement paste matrix. The bonding region or interfacial transition zone (ITZ) in concrete between the matrix and aggregate is a critical component of mechanical performance [19]. For ordinary strength concrete (water-to-binder ratio of 0.5) and low strength concrete (water-to-binder ratio of 0.7), the ITZ is the weak link of concrete, and the compressive strength of concrete is mainly dependent on the strength of the ITZ. When the water-to-cement ratio decreases from 0.7 (low strength concrete) to 0.5 (ordinary strength concrete), the distribution of reaction products in the ITZ becomes more homogeneous [16], and the contribution of 1 gram of CSH to the compressive strength will increase. Hence, when the water-to-cement ratio decreases from 0.7 to 0.5, the strength coefficient *A_1_* increases from 52.39 to 61.54. Alternately, for high strength concrete (water-to-binder ratio of 0.35), the strength of concrete relates to three phases of concrete, *i.e.*, the ITZ phase, bulk paste matrix phase, and aggregate phase. Due to the contribution of the aggregate to the compressive strength, the ratio of the strength of the ITZ to the sum of the other two phases (bulk paste matrix paste plus aggregate phase) will decrease. Thus, when the water-to-cement ratio decreases from 0.5 to 0.3, the strength coefficient *A_1_* also decreases from 61.54 to 55.37.

**Figure 5:**
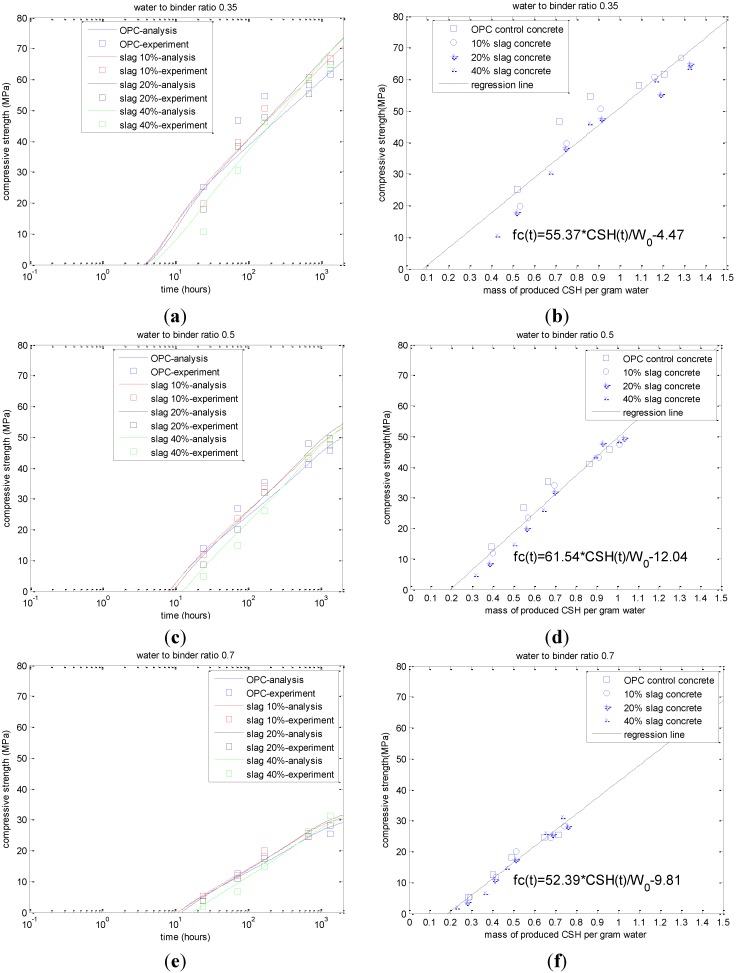
Analysis of the compressive strength development (experimental results are taken from reference [18]). (**a**) compressive strength *versus* age: water-to-binder ratio of 0.35; (**b**) compressive strength *versus* CSH: water-to-binder ratio of 0.35; (**c**) compressive strength *versus* age: water-to-binder ratio of 0.5; (**d**) compressive strength *versus* CSH: water-to-binder ratio of 0.5; (**e**) compressive strength *versus* age: water-to-binder ratio of 0.7; (**f**) compressive strength *versus* CSH: water-to-binder ratio of 0.5.

Figure 5 shows the analysis results for the compressive strength development of slag-blended concrete. First, the proposed model can reflect the effect of the water-to-cement ratio on the compressive strength development of concrete. With the increase in the water-to-cement ratio, given a certain age, the produced CSH content for 1 gram of mixing water will decrease. Hence, the compressive strength will decrease correspondingly; Second, as shown in Figure 5a,c,e, the proposed model can reproduce the strength crossover phenomenon between the control Portland cement concrete and the slag-blended concrete. Because the reactivity of slag is lower than that of Portland cement, the early-age strength of slag-blended concrete is lower than that of the control concrete. However, because the produced CSH content from 1 gram of reacted slag is higher than that from 1 gram of hydrated cement, at late age, for concrete containing 10%, 20%, and 40% slag, the compressive strength of slag-blended concrete can surpass that of the control concrete. In addition, with the increase in the slag replacement ratio, the reactivity of slag will decrease and the age corresponding to the crossover of the compressive strength will be postponed; Third, as shown in Figure 5a,c,e, given certain water-to-binder ratios, with the increase in slag content, the *X*-axis intercept of the strength development function increases correspondingly. This agrees with Brooks *et al.*’s study [20] on the setting time of slag-blended concrete. They reported that the inclusion of slag at replacement levels of 40% and greater resulted in significant retardation in setting times. As the replacement levels of slag were increased, there was greater retardation in setting times. On the other hand, as shown in Figure 5b,d,f, with the increase in the water-to-binder ratio, the *X*-axis intercept of the strength development function will increase. Neville [18] also reported that the setting of concrete increases with an increase in the water-to-binder ratio.

**Figure 6:**
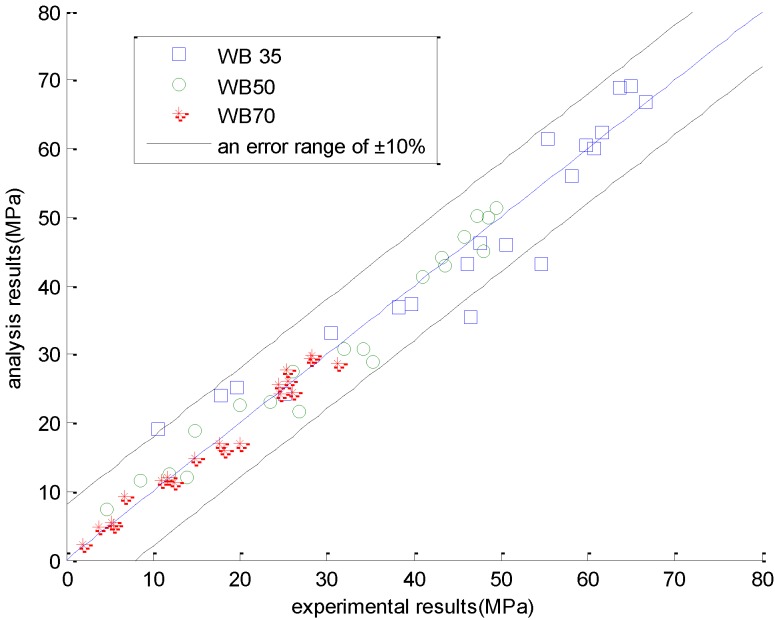
Comparison between the analysis results and experimental results (experimental results are taken from reference [18]).

Figure 6 presents a holistic comparison between the experimental results and the analysis results. As shown in this figure, the analysis results generally agree with the experimental results. For high strength concrete with a water-to-binder ratio of 0.35, due to the ignorance of the aggregate contribution to the compressive strength, the analysis results slightly deviate from the experimental results.

**Figure 7:**
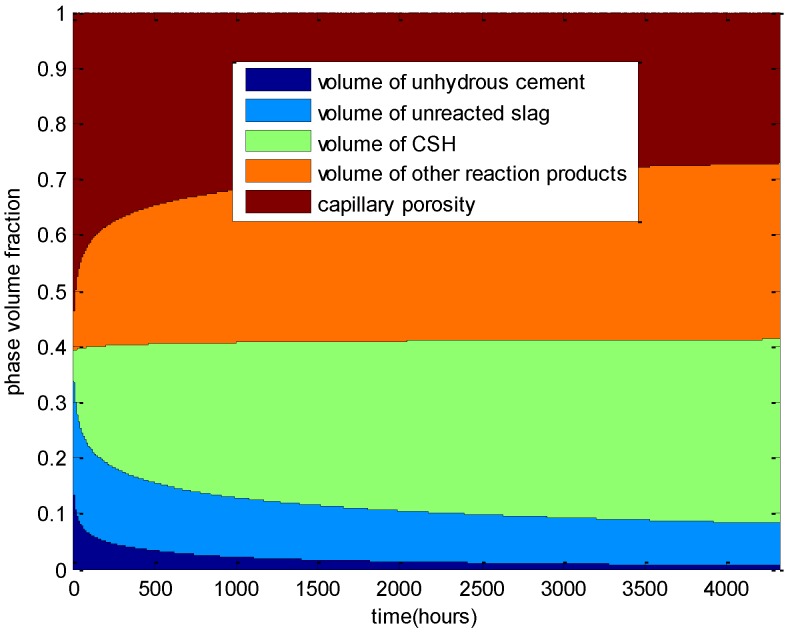
Phase volume fraction evolutions of cement-slag paste: water-to-binder ratio of 0.5, slag replacement ratio of 0.5.

Figure 7 presents the evolution of the phase volume fractions of hardening cement-slag blends (water-to-binder ratio of 0.5 with 50% slag, curing temperature of 20 °C). As shown in this figure, with the progression of cement hydration and slag reaction, the volumes of un-reacted cement and slag decrease, the volumes of CSH and other reaction products increase, and due to the filling effects of the reaction products, the volume of the capillary pore decreases. At an early age, cement hydration and slag reaction proceed quickly, and at a late age, the reaction rates become slower. Because the reactivity of cement is much higher than that of slag, at the age of 180 days, the volume of anhydrous cement is much less than that of unreacted slag.

**Figure 8:**
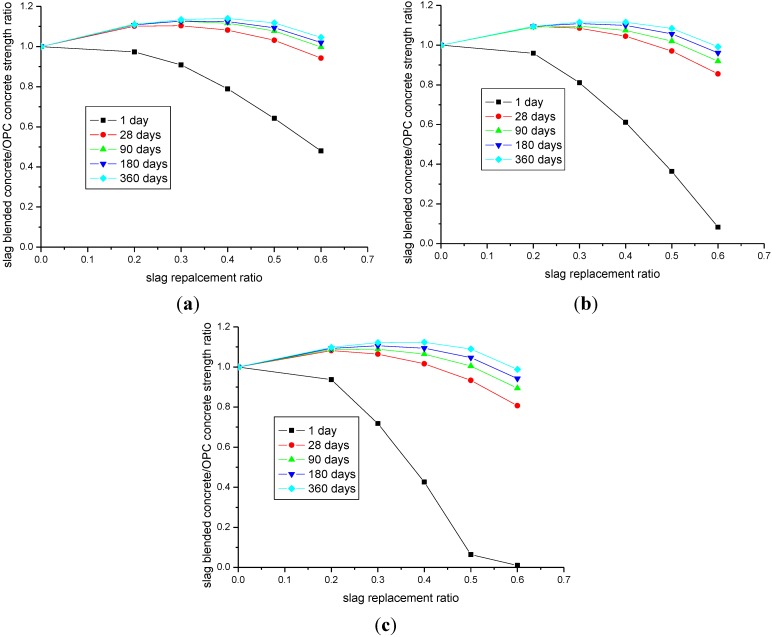
Effects of slag inclusions on the compressive strength development of concrete. (**a**) water-to-binder ratio of 0.35; (**b**) water-to-binder ratio of 0.5; (**c**) water-to-binder ratio of 0.7.

Figure 8 shows the parameter analysis of the effect of slag inclusion on the compressive strength development of concrete. The water-to-binder ratios shown in Figure 8a–c are 0.35, 0.5, and 0.7, respectively. The vertical axis of these figures represents the ratio of the compressive strength between slag-blended concrete and the control Portland cement concrete. As shown in the figures, at the early age of 1 day, with the increase in the slag replacement ratio, the compressive strength of slag-blended concrete almost linearly decreases. As the curing age increases, obviously, the strength of slag-blended concrete with higher slag ratios increases faster, and at a late age, such as 360 days, the maximum value of the strength lies roughly at the slag replacement ratio of 40%. With regard to a slag replacement ratio higher than 40%, due to the lower reactivity of slag (shown in Figure 1), the ultimate strength ratio is less. At the age of 360 days, because of losses in the capillary water, the decrease in available deposition spaces for hydration products, and a change in the hydration rate-determining process to a diffusion-controlled stage, the rate of the reaction becomes much slower, and the change in the compressive strength ratio between slag-blended concrete and OPC concrete is very marginal. Based on the evolution of the compressive strength ratio of concrete with different water-to-binder ratios and different slag inclusions, a slag replacement ratio of 40% can be regarded as the optimum slag content of concrete.

A summary regarding the determination of optimum slag content is as follows: First, using experimental results on the reaction degree of slag, the reaction coefficients of the slag reaction model are calibrated; Second, using experimental results on the compressive strength, the strength development coefficients of slag-blended concrete are calibrated; Third, parameter analysis of the compressive strength development of Portland cement concrete and slag-blended concrete is performed. By comparing the strength development of slag-blended concrete with that of Portland cement concrete, the optimum slag content can be analyzed.

## 4. Conclusions

This paper has proposed a numerical procedure to analyze the optimum usage of slag for the compressive strength of concrete. The conclusions are summarized as follows.

First, we proposed a blended hydration model that simulates cement hydration, slag reaction, interactions between cement hydration and slag reaction, and phase volume fraction evolution of cement–slag blends. The amount of calcium silicate hydrate (CSH), which is closely related to the compressive strength of concrete, is calculated considering the contributions from both cement hydration and slag reaction.

Second, by using a linear equation for the compressive strength and CSH content, the development of the strength of slag-blended concrete is evaluated. Given a certain age, with an increase in the water-to-binder ratio, the compressive strength of concrete will decrease. The proposed model can reproduce the strength crossover phenomenon between the control Portland cement concrete and slag-blended concrete. The early-age strength of slag-blended concrete is lower than that of the control concrete. However, at a late age, for concrete containing 10%, 20%, and 40% slag, the compressive strength of slag-blended concrete can surpass that of the control concrete. With an increase in the slag content and the water-to-binder ratio, the starting age of the compressive strength development will be retarded.

Third, based on parameter analysis, the ratio of the compressive strength between slag-blended concrete and Portland cement concrete is calculated. For concrete with different water-to-binder ratios, at the late age of 360 days, the optimum slag content is approximately 40% of the total binder content. Before this optimum point, the compressive strength of concrete mixtures containing slag increases as the amount of slag increases. After an optimum point, any further increase in slag inclusion does not improve the compressive strength.
